# Effects of rivaroxaban and warfarin on the risk of gastrointestinal bleeding and intracranial hemorrhage in patients with atrial fibrillation: Systematic review and meta‐analysis

**DOI:** 10.1002/clc.23690

**Published:** 2021-07-24

**Authors:** Hongcheng Jiang, Yue Jiang, Haotian Ma, Hesong Zeng, Jiagao Lv

**Affiliations:** ^1^ Division of Cardiology, Department of Internal Medicine, Tongji Hospital, Tongji Medical College Huazhong University of Science and Technology Wuhan Hubei China; ^2^ The First Clinical School, Tongji Medical College Huazhong University of Science and Technology Wuhan Hubei China

**Keywords:** atrial fibrillation, gastrointestinal bleeding, intracranial hemorrhage, rivaroxaban, warfarin

## Abstract

To assess the risk of gastrointestinal bleeding and intracranial hemorrhage in patients with atrial fibrillation (AF) after the use of rivaroxaban or warfarin. To investigate the effects of rivaroxaban and warfarin on gastrointestinal and intracranial hemorrhage in patients with AF, we searched PubMed, Embase, and Medline from the establishment of databases up to 2020. We finally included 38 observational studies involving 1 312 609 patients for the assessment of intracranial hemorrhage, and 33 observational studies involving 1 332 956 patients for the assessment of gastrointestinal bleeding. The rates of intracranial hemorrhage were 0.55% in the rivaroxaban group versus 0.91% in the warfarin group (OR 0.59; 95% CI 0.53–0.66; *p* < .00001, I2 = 78%). The rates of gastrointestinal bleeding were 2.63% in patients with rivaroxaban versus 2.48% in those with warfarin (OR 1.06; 95% CI 0.96–1.17; *p* < .00001, I2 = 94%). Rivaroxaban could significantly reduce the risk of intracranial hemorrhage in patients with AF than warfarin, but the risk of gastrointestinal bleeding remained controversy due to no statistical significant difference. Notably, a subgroup analysis demonstrated that patients in rivaroxaban group with severe chronic renal diseases or undergoing hemodialysis exposed to less gastrointestinal hemorrhage risk than the group from warfarin.

**Abbreviations**
AF: atrial fibrillation
NOACs: nonvitamin K antagonist oral anticoagulants
VKAs: vitamin K antagonists

## 1 INTRODUCTION

Atrial fibrillation (AF), the most prevalent cardiac arrhythmia in clinical practice, is associated with a dramatically increasing risk of ischemic stroke, causes death and disability five‐fold.^1^

Vitamin K antagonists (VKAs), represented by warfarin have been the primary oral anticoagulants for ischemic stroke prevention in AF.^2^ Regarding the former reports, AF was associated with a 60% reduction in the risk of stroke, but also have many deficiencies that have restricted their applies in clinical practice, which was associated with a narrow therapeutic window, multiple interactions with other medications, and therefore there is a demand for real time monitoring for efficacy evaluation and dosage adjustments when use VKAs.^3^

Nowadays non‐VKA oral anticoagulants (NOACs), particularly the factor Xa inhibitors, for example rivaroxaban, have drawn lots of attention considering a number of phase III clinical trials have shown that NOACs are confirmed as effective as VKAs as treatments for the prevention of ischemic stroke or systemic embolism, and have a better safety outcome, especially regarding the risk of major bleeding, which was proved by several net meta‐analysis.^4^, ^5^, ^6^ Although VKAs and NOACs are associated with an obvious reduction in the risk of stroke in AF patients, they have many complications that result in adverse outcomes in clinical practice, including hemorrhage. Several clinical studies have contributed to inquiry of the hemorrhage outcomes in rivaroxaban versus warfarin. Rivaroxaban was the first oral factor Xa inhibitor used to the clinical practice, and provided potential advantages over VKAs, including rapid onset and offset of action, and fewer drug interactions.^7^ It has been already testified for the effect of preventing stroke and systemic embolism. However, the comparative safety outcomes of rivaroxaban and warfarin regimens remain unclear and controversial, especially regarding high‐risk patient groups such as suffering from severe renal diseases, or populations in varied geographical distributions, because of the scarcity and inaccuracy of trials. Thus, we collected data of several observational studies to conduct a meta‐analysis to compare the differences of safety outcomes between rivaroxaban and warfarin.

## 2 METHODS

### 2.1 Search strategy

PubMed, Embase, and medline were systematically searched from the establishment of databases up to 2020. The search strategy was edited to each database and included index terms (medical subject headings) [MeSH] and Emtree) and text words related to AF, rivaroxaban, warfarin and hemorrhage. We also scanned the bibliographies of the included articles and relevant reviews for further references.

### 2.2 Inclusion and exclusion criteria

Cross‐sectional studies, letters to the editor, commentaries/editorials, and previous reviews and meta‐analyses were excluded. Conference abstracts were also excluded as their results are primary and they often contain deficient information causing risk of bias. The primary safety outcomes we regarded as inclusion criteria were gastrointestinal bleeding and intracranial hemorrhage events. (Figure S1 show in the supplemental material.)

### 2.3 Study selection

Two individual reviewers performed study selection. When two individual's screening results are inconsistent, a third person makes the judgment. Titles and abstracts were screened to identify potentially relevant studies and duplicates; all studies identified as potentially relevant by either reviewer proceeded to full‐text review. All the discrepancies were settled by getting through full texture to reach consensus.

### 2.4 Data analysis

Meta‐analytic results were present as adjusted ORs with 95% CIs. The heterogeneity was present with estimation using the I^2^ statistic. All analyses were conducted using Review Manager 5.3.

## 3 RESULTS

**FIGURE 1:**
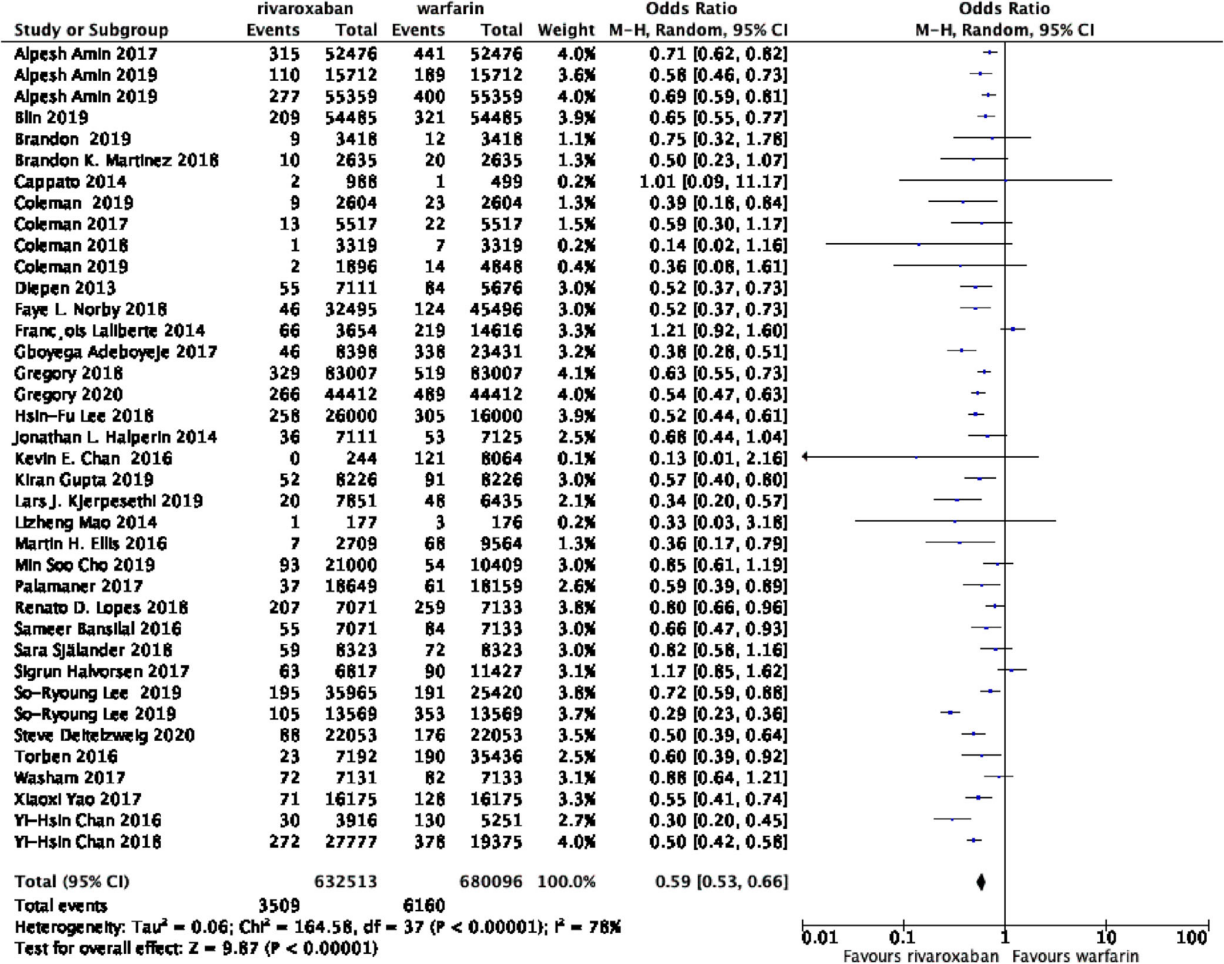
Forest plot of studies assessing the risk of intracranial hemorrhage among patients in the rivaroxaban and warfarin group

**FIGURE 2:**
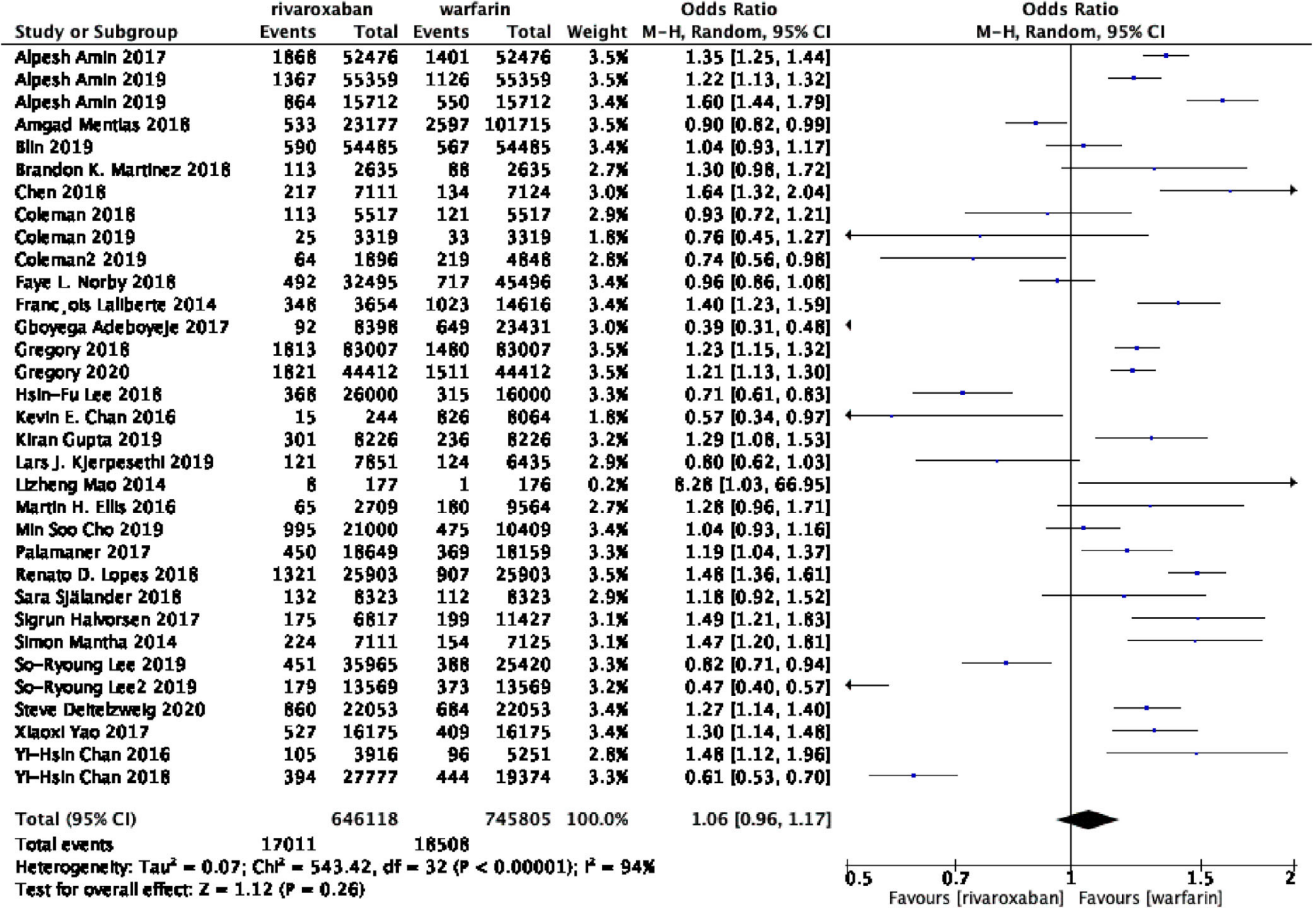
Forest plot of studies assessing the risk of gastrointestinal bleeding among patients in the rivaroxaban and warfarin group

A total of 1778 articles were identified in the initial search. We excluded 122 duplicates and removed 1448 studies not meeting inclusion criteria, and 208 full‐text studies were evaluated in a closer inspection. After 161 articles (three changed drugs during the observational studies, 73 data unavailable, 30 NOAC but not rivaroxaban, five observational studies included patients not only suffered from AF, 50 were only major bleeding and not mentioned gastrointestinal bleeding and intracranial hemorrhage) were discarded, a total of 47 studies were finally included in the analysis. Thirty eight observational studies were included in our review for intracranial hemorrhage, a total of 1 312 609 patients diagnosed with nonvalvular AF, with sample sizes from 353 to 166 014 patients, while 33 observational studies were included in our review for gastrointestinal bleeding, a total of 1 391 923 patients diagnosed with nonvalvular AF, with sample sizes from 353 to 166 014 patients. Figures below (Figure 1 and Figure 2) summarize the main outcomes of the two group of included trials.

### 3.1 Safety outcomes of intracranial hemorrhage between rivaroxaban and warfarin

Data were collected from 38 studies including 632 513 patients in the rivaroxaban group and 680 096 patients in the warfarin group. The rates of intracranial hemorrhage were 0.55% in the rivaroxaban group versus 0.91% in the warfarin group. As is demonstrated in the Figure 1, the risk of intracranial hemorrhage in the rivaroxaban group was significantly lower when compared with the group of warfarin (OR 0.59; 95% CI 0.53–0.66; *p* < .00001, I2 = 78%) However the statistical heterogeneity was high among studies. Thus, we next conducted subgroup analysis.

### 3.2 Subgroup analysis

**FIGURE 3:**
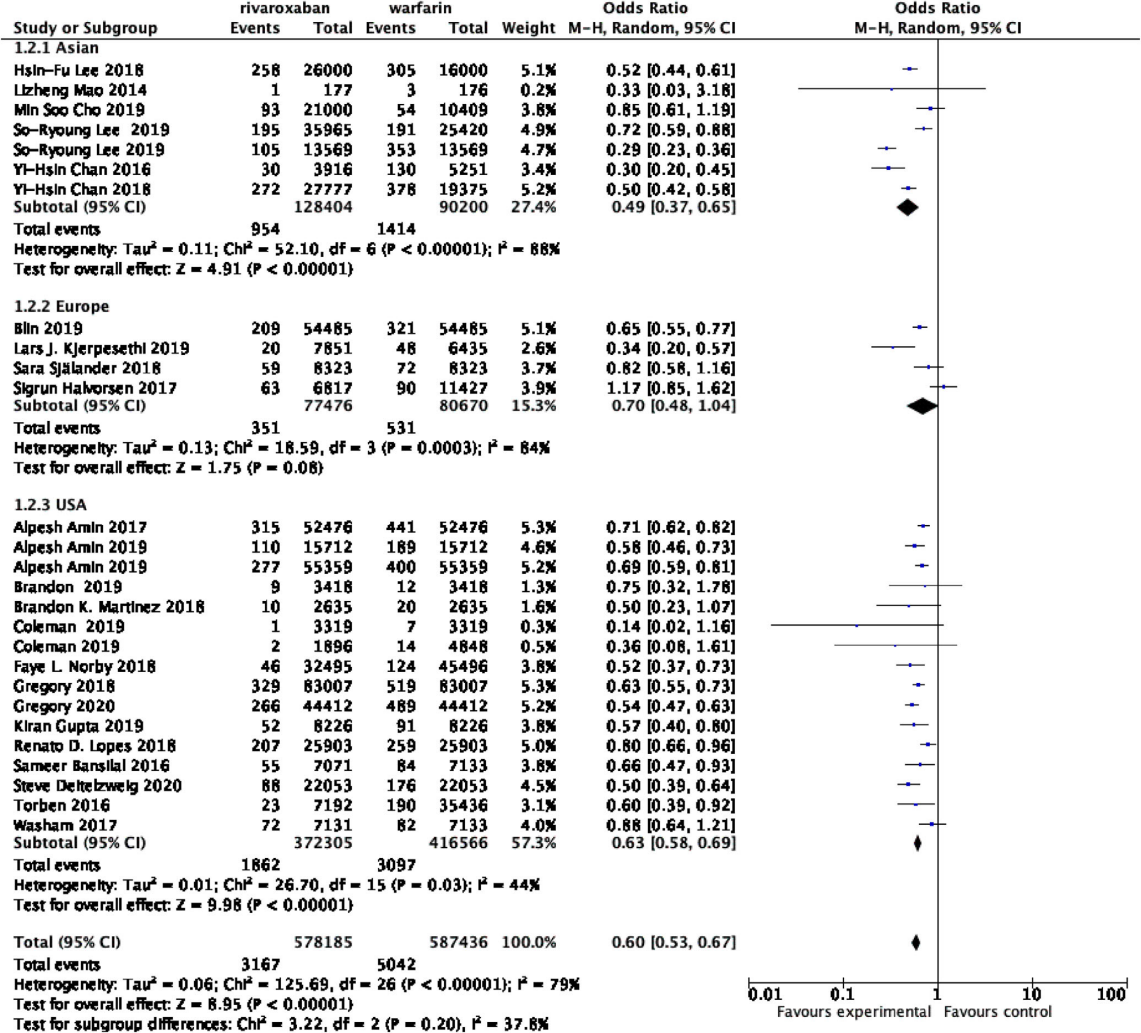
Forrest plot of subgroup analysis assessing the risk of intracranial hemorrhage based on racial distribution

Considering the situation that different patients from varied racial distributions may lead to different outcomes, we conducted subgroup analyses based on 27 trials and divided the data into three subgroups, including 218 604 Asian patients, 788 871 American patients and 158 146 European patients. The rates of intracranial hemorrhage in the rivaroxaban group are 0.74%, 0.50%, 0.45%, respectively. Compared with patients in group rivaroxaban,the rates of warfarin groups are 1.57%, 0.74%, 0.66%, respectively. As was shown in the Figure 3, every subgroup indicated that patients in the rivaroxaban group exposed to obviously lower risk of intracranial hemorrhage than the patients from warfarin group.(Asian: OR 0.49; 95% CI 0.37–0.65; *p*< .00001,I2 = 88%; Europe: OR 0.70;95% CI 0.48–1.04; *p* = .0003,I2 = 84%; USA: OR 0.63;95% CI 0.58–0.69; *p* = .03,I2 = 44%; total: OR 0.60; 95% CI 0.53–0.67; *p*<.00001, I2 = 79%).

### 3.3 Safety Outcomes of gastrointestinal bleeding between rivaroxaban and warfarin

The Figure 2 shows the overall results of the gastrointestinal bleeding outcomes. Data regarding the occurrence of gastrointestinal bleeding are available from 33 trials, 646 118 patients in the group of rivaroxaban and 745 805 patients in the group of warfarin. The rates of gastrointestinal bleeding were 2.63% in patients with rivaroxaban versus 2.48% in those with warfarin. Interestingly, the group of rivaroxaban was associated with similar risk of gastrointestinal bleeding, when compared with the group of warfarin, but the statistical heterogeneity was high among studies. Thus, we next conducted subgroup analysis. (OR 1.06; 95% CI 0.96–1.17; *p* = 0.26, I2 = 94%).

### 3.4 Subgroup analysis

Based on the situation that different patients are under varied healthy conditions and medical treatments, we conducted subgroup analyses on account of chronic renal diseases or undergoing hemodialysis, and racial distribution.

**FIGURE 4:**
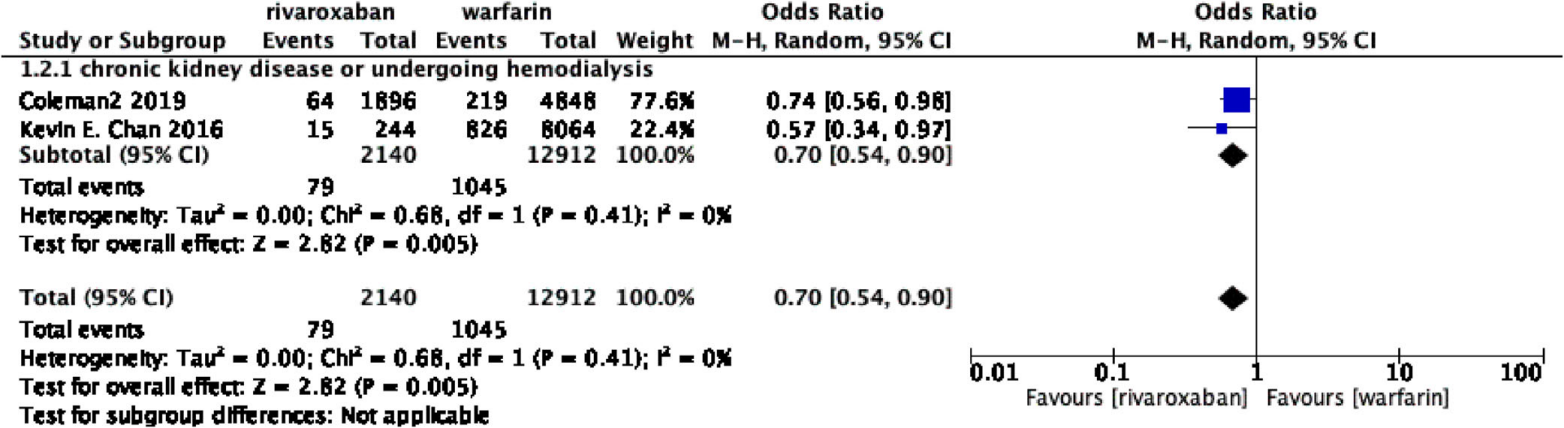
Forrest plot of subgroup analysis assessing the risk of gastrointestinal bleeding considering severe chronic renal diseases or hemodialysis

The outcomes based on two trials showed that the patients from rivaroxaban group suffering from severe chronic renal diseases or undergoing hemodialysis exposed to less gastrointestinal hemorrhage risk than the group from warfarin, including 2140 patients in group rivaroxaban and 12 912 patients in group warfarin, 3.69% versus 8.09%. (OR 0.70; 95% CI 0.54–0.90; *p* = .005, I2 = 0%)(Figure 4).

**FIGURE 5:**
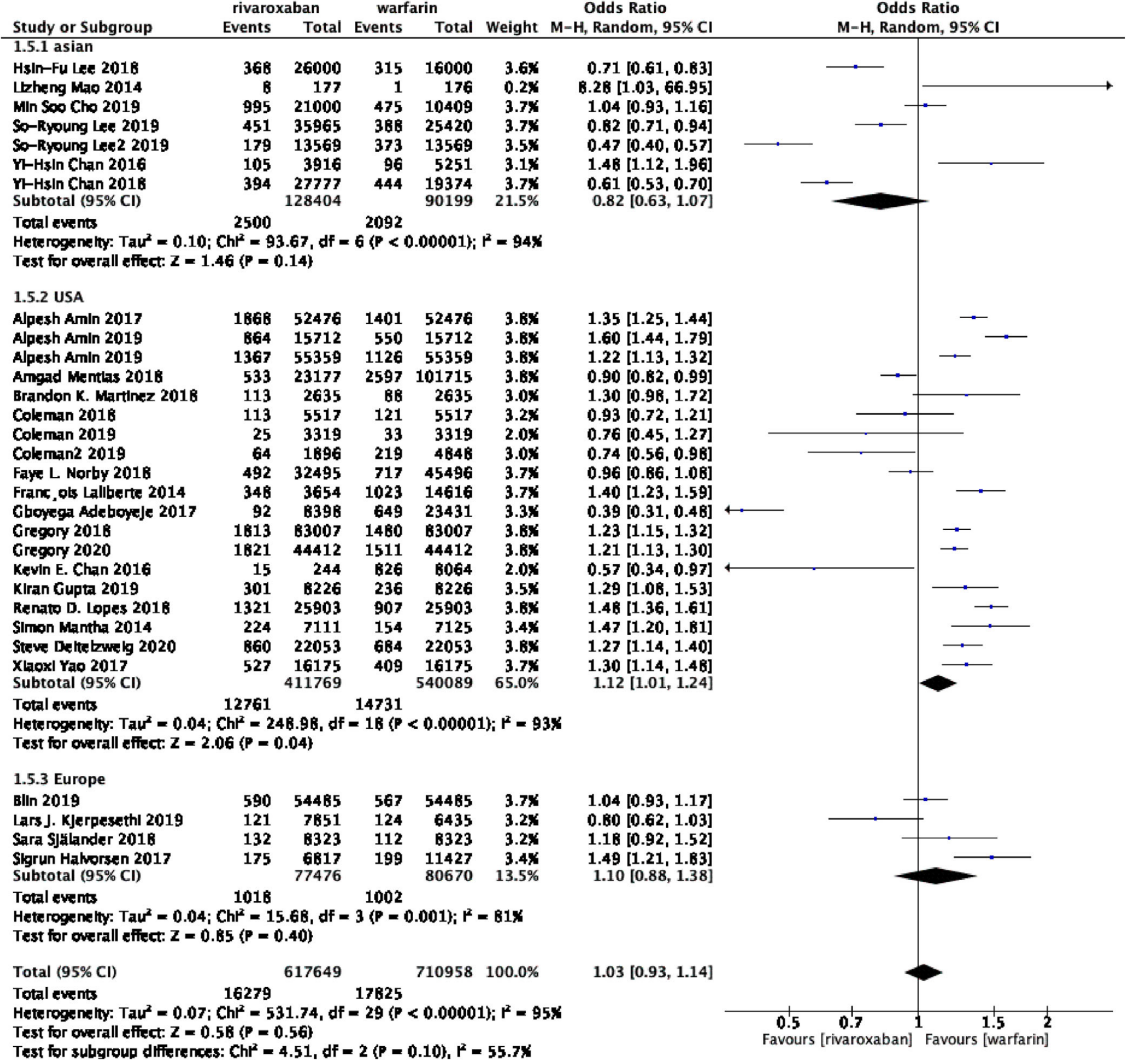
Forrest plot of subgroup analysis assessing the risk of gastrointestinal bleeding based on racial distribution

The outcomes based on 30 trials showed the specific features of patients from varied ethnicities, including Asian, American and European. The data of was collected from 218 603 Asian patients, 951 858 American patients and 158 146 European patients. The rates of gastrointestinal bleeding in the rivaroxaban group are 1.95%, 3.10%, and 1.31%, respectively. Compared with rivaroxaban, the rates of warfarin groups are 2.32%, 2.73%, and 1.24%, respectively. Only data collected from American patients appeared to have the difference of gastrointestinal bleeding risk, between rivaroxaban and warfarin, with 19 studies reporting, demonstrating warfarin results in less gastrointestinal hemorrhage. (USA: OR 1.12; 95% CI1.01–1.24; *p* = .04, I2 = 93%) However it remained uncertain whether the risk of gastrointestinal bleeding between racial variety showed discrepancy since no statistic differences of total events with high statistical heterogeneity. (Asian: OR 0.82; 95% CI 0.63–1.07; *p* < .00001, I2 = 94%; Europe: OR 1.10;95% CI 0.88–1.38; *p* = .001,I2 = 81%; total: OR 1.03; 95% CI 0.92–1.14; *p* = 0.56, I2 = 95%)(Figure 5).

## 4 DISCUSSION

Our meta‐analysis included 38 observational studies involving 1 312 609 patients (632 513 used rivaroxaban and 680 096 used warfarin) with AF, in order to assess the risk of intracranial hemorrhage after warfarin and rivaroxaban use, and 33 observational studies involving 1 391 923 patients (646 118 used rivaroxaban and 745 805 used warfarin) with AF, in order to assess the risk of gastrointestinal bleeding after warfarin and rivaroxaban use.

In our meta‐analysis, we found that anticoagulant therapy with rivaroxaban could significantly reduce the risk of intracranial hemorrhage in patients with AF, which was statistically significant, and partially increased the risk of gastrointestinal bleeding among different ethnic groups of patients, but there was no statistical significance. The reason why rivaroxaban reduces intracranial hemorrhage is not fully understood. A possible explanation could be the disturbance of the hemostasis by inhibition of coagulation factor VIIa, which is highly expressed in brain vessels, forms complexes with tissue factor and is a key initiator of the coagulation cascade,^8^ and rivaroxaban works by inhibiting clotting factor Xa, which is different from warfarin. However, whether rivaroxaban increases gastrointestinal bleeding or not is unclear yet. The reason that rivaroxaban is partially eliminated through the intestine and is a substrate for the P‐glycoprotein transport system could possibly explain why there is a subtle upward trend with no statistic difference.^9^ This system actively pumps drugs into the gastrointestinal tract, allowing it to maintain a higher concentration of the active agent.^10^ In contrast, Warfarin is highly bioavailable and is almost entirely absorbed in the intestinal tract, whereas Warfarin is not bioactive when it is not absorbed.^11^ Anyway, to distinguish the difference of gastrointestinal bleeding risk between two drugs requires more clinical data.

To further investigate the effects of rivaroxaban and Warfarin on bleeding in patients with AF, we performed subgroup analyses based on ethnicity. Not surprisingly, we found that rivaroxaban could significantly reduce the risk of intracranial hemorrhage in patients with AF, regardless of race, although the benefit was more pronounced in Asians. Interestingly, we found that rivaroxaban had a different effect on gastrointestinal bleeding in patients with AF among different ethnic groups, because we found that rivaroxaban could slightly reduce the risk of gastrointestinal bleeding in Asians and slightly increase the risk of gastrointestinal bleeding in Europeans and Americans, but these results were not statistically significant. Thus, to explain the reason for those weak tendency, more data should be collected for further study in order to rule out individual variety. The reason for this phenomenon may be due to the fact that in Asian countries, safety concerns because of the generally lower body mass index of the population^12^, ^13^ in addition to differential bleeding tendencies have resulted in a preference for the underdosed rivaroxaban.^13^

Previous meta‐analysis have shown that rivaroxaban could significantly reduce ischemic events in patients with AF compared to warfarin,^14^ and in our meta‐analysis, we found that rivaroxaban could reduce the risk of intracranial hemorrhage. Meanwhile, rivaroxaban have a more stable INR levels compared with warfarin because it has lower dose–response variability and less interactions.^15^ Besides, Patients with AF may have a better adherence to rivaroxaban than warfarin, likely due to the fact that rivaroxaban do not need constant blood monitoring.^16^ Despite these benefits, it is important to note that rivaroxaban may possibly increase the risk of gastrointestinal bleeding, especially in Europeans and Americans. Even though there is no statistic significance to compare gastrointestinal hemorrhage in varied ethnic groups, but we should regard it with caution to figure out whether this trend makes sense. Moreover, unlike warfarin, rivaroxaban do not currently have a widely available reversal agent and are not routinely monitored with laboratory testing,^7^ which also increase the risk of using rivaroxaban.

Our meta‐analysis also has certain limitation. First of all, we initially intend to analyze the risk of major bleeding, but due to the distinction of different research in the definition of major bleeding, some on the basis of International Society of Thrombosis and Hemostasis, some on the basis of Cunningham algorithm to identify bleeding resulting in the need for hospitalization as a proxy for major bleeding, some did not mention the standard of major bleeding, so we finally analyze and intracranial hemorrhage and gastrointestinal bleeding, which is not controversial in terms of definition. Secondly, our meta‐analysis included observational studies rather than randomized controlled trials, which inevitably led to heterogeneity. At the same time, the enrolled patients in each study are also different in terms of the basic characteristics (gender, age, etc.), basic diseases, basic medication, and AF scores, which may influence the clinic relevant bleeding risk. For instance, patients with cancer are often at an increasing risk for bleeding due to tumor invasion, frequent procedural interventions, endothelial dysfunction,^17^ and elderly patients generally have a higher prevalence of comorbidities and polypharmacy, and a higher risk of bleeding, but lower mobility for frequent laboratory monitoring,^18^, ^19^ It is to be noted that renal impairment is an independent risk factor for bleeding in AF patients.^20^, ^21^ As shown in Figure 4, patients in rivaroxaban group with severe chronic renal diseases or undergoing hemodialysis exposed to less gastrointestinal hemorrhage risk than the group from warfarin. It has to be mentioned half of the rivaroxaban in the body need to be metabolized by kidney. Normal renal function could help the body to minimize the accumulation of rivaroxaban and reduce the side effect of drugs. However, cumulative medication toxicity could not be cleared by body who suffering from renal dysfunction which accounts for elevated hemorrhage rick. But warfarin is different because of its metabolic pathway, which mostly depends on hepatic metabolism. Finally, the dose of rivaroxaban in different studies is not completely the same. Some studies may use 20 mg, some studies use 15 mg due to renal insufficiency of the included patients, and some experiments do not mention the dose of rivaroxaban, which may also affect the results of the experiments.

## 5 CONCLUSION

Rivaroxaban could significantly reduce the risk of intracranial hemorrhage in patients with AF, but the risk of gastrointestinal bleeding remained controversy due to no statistical significant difference. Notably, a subgroup analysis demonstrated that patients in rivaroxaban group with severe chronic renal diseases or undergoing hemodialysis exposed to less gastrointestinal hemorrhage risk than the group from warfarin.

## CONFLICT OF INTEREST

The authors declare that there is no conflict of interest.

## Supporting information

**Figure S1**: Flow charts showing relevant studies

## Data Availability

All data included in this study are available upon request by contact with the corresponding author.
