# Supplementary figures and images for: Effects of rivaroxaban and warfarin on the risk of gastrointestinal bleeding and intracranial hemorrhage in patients with atrial fibrillation: Systematic review and meta‐analysis
Source: Clin Cardiol. 2021 Jul 24;44(9):1208–15. doi: 10.1002/clc.23690 (PMC8427974; doi:10.1002/clc.23690)

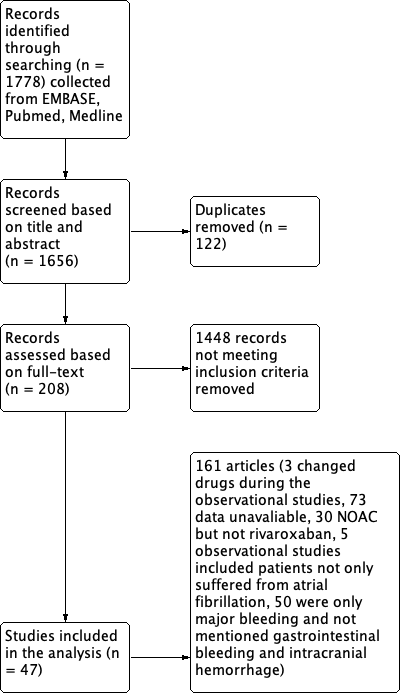

Supplement: Supplementary file 1 — Figure S1: Flow charts showing relevant studies [file CLC-44-1208-s001.tif]
